# Impact of Low- Versus Standard-Pressure Pneumoperitoneum on Postoperative Recovery in Patients with Obesity Undergoing Robot-Assisted Radical Prostatectomy: A Retrospective Cohort Study

**DOI:** 10.3390/medicina61071253

**Published:** 2025-07-10

**Authors:** Resul Sobay, Hasan Samet Güngör, Abdurrahman İnkaya, Murat Beyatlı, Ahmet Tahra, Eyüp Veli Küçük

**Affiliations:** Department of Urology, Umraniye Training and Research Hospital, 34764 Istanbul, Turkey; drsametgngr@gmail.com (H.S.G.); ainkaya@hotmail.com (A.İ.); muratbeyatli_90@hotmail.com (M.B.); ahmettahra@gmail.com (A.T.); eyupveli@gmail.com (E.V.K.)

**Keywords:** obesity, pneumoperitoneum, quality of recovery, robot-assisted prostatectomy, surgical workspace

## Abstract

*Background and Objectives*: Low-pressure pneumoperitoneum (PP) during robot-assisted radical prostatectomy (RARP) has been shown to improve postoperative recovery in patients with non-obesity. However, its efficacy in individuals with obesity remains unclear. This study aimed to evaluate postoperative outcomes in patients with obesity undergoing RARP with low-pressure (7 mmHg) versus standard-pressure (12 mmHg) PP. *Materials and Methods*: In this retrospective cohort study, 130 patients with obesity (BMI > 30 kg/m^2^) undergoing RARP were divided into low-pressure (*n* = 60) and standard-pressure (*n* = 70) groups. Postoperative recovery was assessed using the Quality of Recovery-15 (QoR-15) questionnaire on postoperative days (POD) 1, 3, and 30. Secondary outcomes included surgical workspace (SWS) scores, operative time, blood loss, intraoperative and postoperative complications, hospital stay, and pathological results. ANCOVA and chi-square tests were used for analysis. *Results*: QoR-15 scores were significantly higher in the low-pressure group on POD1 (123.58 vs. 111.41), POD3 (128.37 vs. 116.41), and POD30 (132.88 vs. 125.61; *p* < 0.001). Operative time (98.5 vs. 71.57 min; *p* < 0.001) and blood loss (129 vs. 97.07 mL; *p* = 0.039) were higher in the low-pressure group. SWS scores were lower in the low-pressure group (*p* < 0.001). There were no significant differences between groups in complication rates, hospital stay, or positive surgical margins. *Conclusions*: In patients with obesity undergoing RARP, low-pressure PP improves postoperative recovery without increasing complications. Despite longer operative times and higher blood loss, this approach is a viable option to enhance recovery in this population.

## 1. Introduction

Prostate cancer is a prevalent malignancy among men, and robot-assisted radical prostatectomy (RARP) is widely used for localized disease due to its precision and lower morbidity compared with open surgery [1]. During RARP, standard pneumoperitoneum (PP) pressure (12–15 mmHg) ensures adequate surgical workspace. However, this may exacerbate cardiopulmonary and inflammatory responses, particularly in patients with obesity [2]. Obesity—an increasingly prevalent comorbidity among patients with prostate cancer—reduces abdominal compliance and increases surgical risk [3]. Patients with obesity undergoing RARP face unique challenges including altered anatomical landmarks, increased visceral fat, reduced abdominal compliance, and heightened risk of perioperative complications. These factors significantly impact surgical visualization, workspace adequacy, and postoperative recovery trajectories [4]. Recent studies have highlighted the correlation between BMI and prostate volume, which significantly affects surgical complexity and workspace quality [5].

While low-pressure PP (6–8 mmHg) has been shown to improve recovery in patients with non-obesity, its efficacy in the obese population remains underexplored [6]. This study aimed to investigate whether low-pressure PP (7 mmHg) improves postoperative quality of recovery compared with standard-pressure PP (12 mmHg) in patients with obesity undergoing RARP, using the QoR-15 questionnaire and intraoperative outcomes.

## 2. Materials and Methods

This retrospective cohort study was conducted at Ümraniye Training and Research Hospital, a tertiary referral center in Istanbul, Turkey. One hundred and thirty male patients with obesity (BMI > 30 kg/m^2^) who underwent RARP between January 2023 and December 2024 were included. Patient assignment to low-pressure versus standard-pressure groups was based on chronological periods: standard-pressure procedures were performed from January 2023 to June 2023, while low-pressure procedures were performed from July 2023 to December 2024. Patients were divided into two groups based on PP pressure: low-pressure (7 mmHg, *n* = 60) and standard-pressure (12 mmHg, *n* = 70). This study was approved by the Ethics Committee of University of Health Sciences Ümraniye Training and Research Hospital(Approval No: 2025/58, Approval Date: 13 March 2025).

Inclusion criteria were histologically confirmed prostate cancer and BMI > 30 kg/m^2^. Exclusion criteria included prior pelvic radiotherapy or inability to complete the QoR-15 questionnaire.

The primary outcome was the postoperative quality of recovery, assessed using the 15-item Quality of Recovery-15 questionnaire (QoR-15) on postoperative days (POD) 1, 3, and 30. The QoR-15 evaluates five domains: pain, physical comfort, physical independence, psychological support, and emotional state, with scores ranging from 0 to 150 [5]. Secondary outcomes included surgical workspace (SWS) score (rated 3–5 by the surgeon), operative time (minutes), intraoperative blood loss (mL), number of retrieved lymph nodes, postoperative complications (graded using the Clavien–Dindo classification), length of hospital stay (days), and histopathological outcomes (pathological stage, ISUP grade, margin status).

All procedures were performed using the da Vinci Surgical System (Intuitive Surgical, Sunnyvale, CA, USA) by a surgeon with experience in over 1000 RARP cases. PP pressure was set using a CO_2_ insufflator (ENDOFLATOR; Karl Storz, Tuttlingen, Germany).

Data were collected from electronic medical records. QoR-15 questionnaires were administered in person on POD1 and POD3, and via telephone on POD30.

The sample size was calculated to detect a clinically meaningful 8-point difference in QoR-15 scores between low- and standard-pressure groups, based on the minimal clinically important difference (MCID) for QoR-15 [7]. Assuming a standard deviation of 15, 80% power, and 5% type I error (two-tailed), approximately 56 patients per group were required. To accommodate potential data loss in the retrospective design (estimated at 20%), the target sample size was set at 140 patients.

Normality was assessed using the Shapiro–Wilk test. Continuous variables were compared using independent *t*-tests or ANOVA; categorical variables were analyzed using chi-square, Fisher–Freeman–Halton, or Fisher’s exact tests. Analysis of covariance (ANCOVA) was used to adjust QoR-15 scores for BMI. Missing data (<5%) were handled with listwise deletion. The randomness of missing data was verified using the Shapiro–Wilk test (*p* > 0.05). A *p*-value < 0.05 was considered significant. All analyses were performed using SPSS v.28 (IBM Corp., Armonk, NY, USA).

## 3. Results

Baseline characteristics were comparable between the groups in terms of age, PSA level, prostate volume, hypertension, diabetes mellitus, prior abdominal surgery, clinical T stage, and ISUP grade (*p* > 0.05; Table 1). However, the low-pressure group had a significantly higher mean BMI (33.32 ± 2.42 vs. 32.16 ± 1.33 kg/m^2^; *p* = 0.001).

QoR-15 data were missing for three patients (2.1%) on POD1, two patients (1.4%) on POD3, and five patients (3.5%) on POD30, primarily due to incomplete questionnaire responses. As the missing rate was low and confirmed to be random (Shapiro–Wilk test, *p* > 0.05), these cases were excluded using listwise deletion. Operative time and blood loss data were completed for all patients. A total of 130 patients (60 in the low-pressure group and 70 in the standard-pressure group) were included in the final analysis.

**Figure 1:**
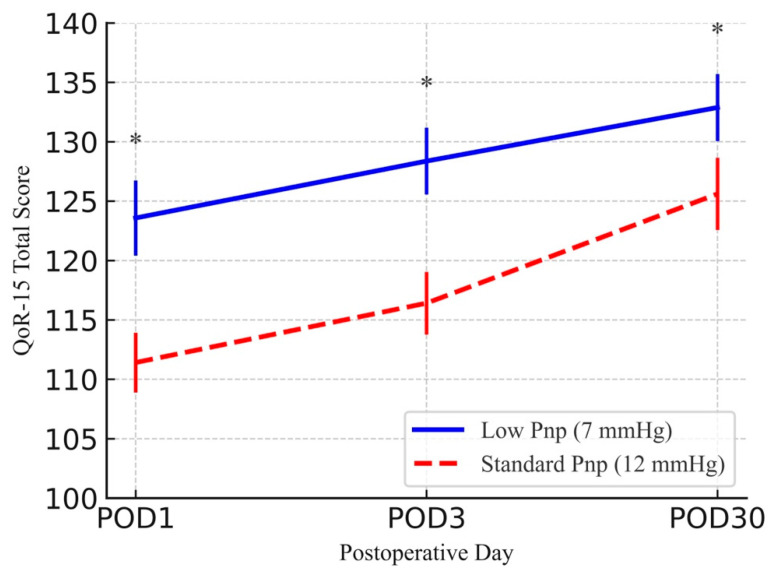
Comparison of QoR-15 total scores at POD1, POD3, and POD30 between low-pressure (7 mmHg) and standard-pressure (12 mmHg) pneumoperitoneum groups. Error bars represent mean ± standard deviation. * *p* < 0.001, ANCOVA (BMI used as covariate).

The low-pressure group exhibited significantly higher QoR-15 scores at POD1 (123.58 ± 3.16 vs. 111.41 ± 2.52), POD3 (128.37 ± 2.81 vs. 116.41 ± 2.64), and POD30 (132.88 ± 2.82 vs. 125.61 ± 3.04; all *p* < 0.001; Figure 1). Domain-specific analysis revealed improvements in pain, physical comfort, psychological support, and emotional state across all time points (*p* < 0.001), while physical independence differed only on POD3 (*p* = 0.005; Table 2).

Among patients who underwent lymph node dissection, the mean number of retrieved lymph nodes was comparable between groups (low-pressure: 12.8 ± 4.2 vs. standard-pressure: 13.1 ± 3.8; *p* = 0.697).

Operative time was significantly longer in the low-pressure group (98.5 ± 16.5 vs. 71.57 ± 12.02 min; *p* < 0.001), and intraoperative blood loss was higher (129 ± 108.73 vs. 97.07 ± 48.4 mL; *p* = 0.039; Table 3). SWS scores were lower in the low-pressure group, with 53.3% scoring SWS 3 compared with 0% in the standard-pressure group (*p* < 0.001). Patients with SWS 3 had higher BMI (*p* = 0.015) and a history of abdominal surgery (*p* = 0.004).

No differences were observed between the groups in terms of hospital stay (2.3 ± 0.83 vs. 2.09 ± 0.33 days; *p* = 0.064), rate of lymph node dissection (50.0% vs. 44.3%; *p* = 0.515), positive surgical margins (13.3% vs. 8.6%; *p* = 0.383), postoperative transfusion rates (5% vs. 1.4%; *p* = 0.335), or Clavien–Dindo complication grades (*p* = 0.185). The low-pressure group had a higher proportion of pT2a (23.3% vs. 8.6%) and ISUP grade 1 (65.0% vs. 47.1%) tumors, while the standard-pressure group had more pT2c (65.7% vs. 45.0%) and ISUP grade 2 tumors (18.6% vs. 6.7%; *p* = 0.017 and *p* = 0.037, respectively; Table 3).

## 4. Discussion

This study is the first to evaluate low-pressure (7 mmHg) versus standard-pressure (12 mmHg) PP during RARP in patients with obesity (BMI > 30 kg/m^2^), a population with unique anatomical and physiological challenges. Our findings demonstrate that low-pressure PP significantly improves postoperative quality of recovery, as measured by the QoR-15 questionnaire, on POD 1, 3, and 30, without an increase in complication rates (Figure 1). However, longer operative times, increased intraoperative blood loss, and reduced SWS scores highlight trade-offs that require careful consideration [1,2]. These results position low-pressure PP as a transformative strategy for optimizing outcomes in high-risk patients in line with enhanced recovery after surgery (ERAS) principles [8].

The superior QoR-15 scores in the low-pressure group, particularly in pain, physical comfort, psychological support, and emotional state domains, suggest that lower insufflation pressure may mitigate the physiological stress of laparoscopy. Preclinical studies indicate that higher insufflation pressure induces peritoneal stretch, triggering the release of pro-inflammatory cytokines (e.g., IL-6, TNF-α) and oxidative stress that exacerbate postoperative pain and delayed recovery [9,10]. In patients with obesity, who already exhibit baseline inflammation due to adipose tissue, low-pressure PP likely attenuates these responses, which may explain the sustained improvements in QoR-15 scores observed in our cohort [11]. Notably, physical independence did not differ at POD1 and POD30, possibly due to the limited sensitivity of this domain in early recovery or the confounding effect of obesity-related mobility constraints [12]. These findings extend prior evidence from non-obese populations, where low-pressure PP improved early pain outcomes but lacked long-term assessment [13].

The significant BMI difference between our study groups (33.32 vs. 32.16 kg/m^2^) represents a potential confounder that may have influenced our results. Higher BMI is associated with increased abdominal wall thickness, altered respiratory mechanics, and greater inflammatory burden, all of which could independently affect recovery outcomes [14]. While we used ANCOVA to adjust for BMI differences, residual confounding cannot be entirely excluded, and this limitation should be considered when interpreting our findings.

Our results align with randomized trials and meta-analyses in non-obese RARP cohorts. For instance, Alhusseinawi et al. reported a 10-point QoR-15 improvement with low-pressure PP (8 mmHg) but excluded patients with BMI > 30 kg/m^2^ [6]. Similarly, Ferroni and Abaza demonstrated reduced pain with ultra-low-pressure PP (6 mmHg), yet their cohort was limited to patients with non-obesity [13]. A meta-analysis by El-Taji et al. found no differences in complications or oncologic outcomes between low- and standard-pressure PP, consistent with our data [15]. Unlike these studies, our focus on patients with obesity addresses a critical gap, as obesity increases laparoscopic complexity due to reduced abdominal compliance and excess visceral fat [3]. The absence of increased complications in our low-pressure group refutes concerns that reduced workspace compromises surgical safety, a hypothesis raised in the earlier laparoscopic literature [16].

The increased operative time (mean difference: 26.93 min) and blood loss (mean difference: 31.93 mL) in the low-pressure group reflect the reduced tamponade effect on venous bleeding and a narrower visual field, necessitating meticulous dissection [17]. These findings are consistent with those of Rohloff et al., who noted a 20 min increase in operative time with low-pressure PP [18]. However, the lack of increased transfusion rates or prolonged hospital stays suggests that these differences are clinically negligible [19]. Surgeons adopting low-pressure techniques should be aware of these challenges, particularly in patients with BMI > 35 kg/m^2^ or prior abdominal surgeries, who exhibited lower SWS scores in our cohort [20]. Training in advanced visualization techniques or using high-definition imaging systems may mitigate these limitations [21].

The substantial proportion of patients (53.3%) with an SWS score of 3 in the low-pressure group raises important concerns about the technical feasibility and generalizability of this approach. An SWS score of 3 indicates significantly compromised surgical workspace, which may limit the technique’s applicability, particularly in less experienced centers or in patients with higher BMI or previous abdominal surgery. This finding suggests that low-pressure pneumoperitoneum should be implemented with careful patient selection and adequate surgical expertise [22].

The comparable lymph node retrieval rates between groups (12.8 vs. 13.1 nodes) provide reassurance that oncologic adequacy is maintained despite reduced workspace. This finding suggests that, despite the reduced surgical workspace in the low-pressure group, lymph node identification and dissection were not significantly compromised, possibly due to the surgeon’s experience. Future implementation of low-pressure techniques might benefit from the routine incorporation of enhanced visualization techniques like free-indocyanine green and fluorescence imaging, particularly in patients with obesity where anatomical identification can be challenging [23,24].

Despite reduced SWS, oncologic outcomes, including positive surgical margins (13.3% vs. 8.6%), were comparable between groups, affirming the oncologic safety of low-pressure PP [25]. The higher proportion of pT2a and ISUP 1 tumors in the low-pressure group, contrasted with the greater frequency of pT2c and ISUP 2 in the standard-pressure group, may reflect selection bias or underlying differences in tumor biology [26]. Patients with obesity often present with less aggressive prostate cancer, potentially explaining these histopathological variations [27]. The higher proportion of lower grade tumors in the low-pressure group may reflect selection bias or temporal changes in patient characteristics over the study period. Future prospective studies should include comprehensive preoperative imaging with multiparametric MRI and standardized biopsy protocols to minimize such bias [28]. Future studies should explore whether low-pressure PP influences pathological staging through altered intraoperative dynamics, such as reduced tissue manipulation [29].

The integration of low-pressure PP into ERAS protocols offers a patient-centered approach, particularly for patients with obesity at risk of delayed recovery [8]. Clinically, it should be prioritized for patients with high BMI and low-risk prostate cancer, where extended operative times pose minimal risk. Experienced surgical teams may incorporate this technique with minimal workflow disruption, although those with less robotic experience may require additional training to maintain adequate SWS [30]. Combining low-pressure PP with multimodal analgesia and early mobilization could further enhance recovery according to ERAS principles [31].

Our study had several limitations. First, its retrospective design introduces the possibility of selection bias, as low-pressure PP may have been selectively applied to patients with favorable anatomical characteristics. Although adjusted statistically, the higher mean BMI in the low-pressure group may have exaggerated SWS and operative time differences [32]. Second, our relatively small sample size of 130 patients, while adequately powered for the primary outcome, may limit the generalizability of our findings and reduce the ability to detect smaller but clinically relevant differences in secondary outcomes [33]. Third, we did not assess inflammatory biomarkers (e.g., C-reactive protein), limiting mechanistic insight, though the literature suggests low-pressure PP reduces peritoneal trauma [10]. Fourth, long-term functional outcomes such as urinary continence and erectile function were not assessed, representing a critical limitation. These outcomes are paramount in prostate cancer surgery and should be prioritized in future prospective studies to provide a comprehensive evaluation of low-pressure pneumoperitoneum benefits [34]. Finally, the substantial proportion of patients with poor surgical workspace (SWS 3) in the low-pressure group raises concerns about the technique’s feasibility and reproducibility across different surgical settings and experience levels [35]. Our study was conducted at a high-volume center, potentially limiting generalizability to community settings with less experienced teams [26].

Despite these limitations, our study offers novel and clinically relevant insights. Given the rising global obesity prevalence and the increasing use of robotic techniques, our findings support the use of low-pressure PP in patients with obesity undergoing RARP. Future studies should assess long-term oncologic and functional outcomes to validate the durability of its benefits. In an era of rising obesity, low-pressure PP has the potential to redefine patient-centered outcomes in robotic surgery, offering a scalable strategy to enhance recovery while addressing the challenges of high-risk populations.

## 5. Conclusions

In conclusion, low-pressure PP (7 mmHg) during RARP significantly improves postoperative recovery in patients with obesity without compromising oncologic outcomes. Despite longer operative times and increased blood loss, its integration into ERAS protocols appears feasible and beneficial for this high-risk patient population. However, the substantial proportion of patients with compromised surgical workspace and the potential for selection bias warrant careful consideration and further investigation through randomized controlled trials. Implementation should be accompanied by appropriate patient selection, surgical expertise, and consideration of enhanced visualization techniques to optimize outcomes.

## Figures and Tables

**Table 1:** Baseline characteristics.

Parameter	Standard-Pressure PP (*n* = 70)	Low-Pressure PP (*n* = 60)	*p* *
Age, years, mean ± SD	63.39 ± 6.4	62.65 ± 5.97	0.502
BMI, kg/m^2^, mean ± SD	32.16 ± 1.33	33.32 ± 2.42	**0.001**
PSA, ng/mL, mean ± SD	7.98 ± 4.33	8.73 ± 4.98	0.359
Prostate volume, mL, mean ± SD	57.21 ± 33	58.5 ± 29.47	0.816
Hypertension, *n* (%)	30 (42.9)	34 (56.7)	0.116
Diabetes mellitus, *n* (%)	36 (51.4)	29 (48.3)	0.725
Prior abdominal surgery, *n* (%)	8 (11.4)	10 (16.7)	0.389
Clinical T stage, *n* (%)			0.273
T2a	28 (40.0)	27 (45.0)	
T2b	11 (15.7)	4 (6.7)	
T2c	31 (44.3)	29 (48.3)	
Clinical ISUP grade, *n* (%)			0.181
1	38 (54.3)	42 (70.0)	
2	8 (11.4)	3 (5.0)	
3	17 (24.3)	13 (21.7)	
4	7 (10.0)	2 (3.3)	

BMI—body mass index, PSA—prostate-specific antigen, ISUP—International Society of Urological Pathology. * Independent samples *t*-test, Pearson’s chi-square test, Fisher–Freeman–Halton exact test. Significant *p* values are written in bold.

**Table 2:** Comparison of QoR-15 scores on POD1, POD3, and POD30.

Category	Time Point	Standard-Pressure PP (Mean ± SD)	Low-Pressure PP (Mean ± SD)	Difference (95% CI)	*p* *
QoR-15 total	POD1	111.41 ± 2.52	123.58 ± 3.16	12.17 (11.16, 13.17)	**<0.001**
QoR-15 total	POD3	116.41 ± 2.64	128.37 ± 2.81	11.95 (11.01, 12.90)	**<0.001**
QoR-15 total	POD30	125.61 ± 3.04	132.88 ± 2.82	7.27 (6.25, 8.29)	**<0.001**
Pain	POD1	13.33 ± 0.85	15.60 ± 0.81	2.27 (1.98, 2.56)	**<0.001**
Pain	POD3	14.46 ± 1.03	17.38 ± 0.76	2.93 (2.61, 3.24)	**<0.001**
Pain	POD30	15.89 ± 0.84	18.22 ± 0.61	2.33 (2.07, 2.59)	**<0.001**
Physical comfort	POD1	38.10 ± 1.48	42.85 ± 1.56	4.75 (4.22, 5.28)	**<0.001**
Physical comfort	POD3	39.97 ± 1.47	44.63 ± 1.29	4.66 (4.18, 5.15)	**<0.001**
Physical comfort	POD30	43.40 ± 1.95	45.93 ± 1.33	2.53 (1.96, 3.11)	**<0.001**
Physical independence	POD1	12.27 ± 1.01	12.38 ± 1.29	0.11 (−0.29, 0.51)	0.296
Physical independence	POD3	13.70 ± 0.98	14.12 ± 1.01	0.42 (0.07, 0.76)	**0.005**
Physical independence	POD30	15.83 ± 1.08	15.80 ± 1.02	−0.03 (−0.39, 0.34)	0.622
Psychological support	POD1	18.63 ± 0.68	19.27 ± 0.71	0.64 (0.40, 0.88)	**<0.001**
Psychological support	POD3	18.44 ± 0.71	19.18 ± 0.70	0.74 (0.49, 0.99)	**<0.001**
Psychological support	POD30	18.60 ± 0.71	19.12 ± 0.72	0.52 (0.27, 0.76)	**<0.001**
Emotional state	POD1	29.09 ± 1.35	33.48 ± 1.85	4.40 (3.83, 4.97)	**<0.001**
Emotional state	POD3	29.84 ± 1.50	33.05 ± 2.14	3.21 (2.55, 3.86)	**<0.001**
Emotional state	POD30	31.90 ± 1.47	33.82 ± 1.73	1.92 (1.35, 2.48)	**<0.001**

QoR—quality of recovery, POD—postoperative day. * ANCOVA (BMI as covariate). Significant *p* values are written in bold.

**Table 3:** Secondary surgical outcomes.

Outcome	Standard-Pressure PP (*n* = 70)	Low-Pressure PP (*n* = 60)	*p* *
Console time, min, mean ± SD	71.57 ± 12.02	98.5 ± 16.5	**<0.001**
Hospital stay, days, mean ± SD	2.09 ± 0.33	2.3 ± 0.83	0.064
Intraoperative blood loss, mL, mean ± SD	97.07 ± 48.4	129 ± 108.73	**0.039**
Pathological T stage, *n* (%)			**0.017**
pT2a	6 (8.6)	14 (23.3)	
pT2b	0 (0)	3 (5.0)	
pT2c	46 (65.7)	27 (45.0)	
pT3a	17 (24.3)	15 (25.0)	
pT3b	1 (1.4)	1 (1.7)	
ISUP grade, *n* (%)			**0.037**
1	33 (47.1)	39 (65.0)	
2	13 (18.6)	4 (6.7)	
3	12 (17.1)	12 (20.0)	
4	12 (17.1)	4 (6.7)	
5	0 (0)	1 (1.7)	
Lymph node dissection, *n* (%)	31 (44.3)	30 (50.0)	0.515
Positive surgical margin, *n* (%)	6 (8.6)	8 (13.3)	0.383
Clavien–Dindo complications, *n* (%)			0.185
0	57 (81.4)	42 (70)	
1	8 (11.4)	7 (11.7)	
2	5 (7.1)	9 (15.0)	
3a	0 (0)	2 (3.3)	
Postoperative transfusion, *n* (%)	1 (1.4)	3 (5.0)	0.335
SWS, *n* (%)			**<0.001**
3	0 (0)	32 (53.3)	
4	27 (38.6)	28 (46.7)	
5	43 (61.4)	0 (0)	

SWS—surgical workspace. * Independent samples t-test, Pearson’s chi-square test, Fisher–Freeman–Halton exact test, Fisher’s exact test. Significant *p* values are written in bold.

## Data Availability

The data presented in this study are available on request from the corresponding author.

## Abbreviations

The following abbreviations are used in this manuscript:

BMI	Body mass index
ERAS	Enhanced recovery after surgery
MCID	Minimal clinically important difference
ISUP	International Society of Urological Pathology.
QOR	Quality of recovery
POD	Postoperative day
PP	Pneumoperitoneum
PSA	Prostate specific antigen
RARP	Robot-assisted radical prostatectomy
SWS	Surgical workspace
